# Mucosa-associated lymphoid tissue lymphoma of the duodenum together with multiple intra-abdominal thromboses and hepatitis C virus infection: a case report

**DOI:** 10.1186/1757-1626-2-9354

**Published:** 2009-12-18

**Authors:** Abdullah Ozkok, Fatih Tufan, Sule Namli, Mesut Bulakci, Binnur Pinarbasi, Oner Dogan, Mehmet Akif Karan, Cemil Tascioglu

**Affiliations:** 1 Department of Internal Medicine, Istanbul University, Istanbul Faculty of Medicine, Istanbul (34390), Turkey; 2 Department of Radiodiagnostics, Istanbul University, Istanbul Faculty of Medicine, Istanbul (34390), Turkey; 3 Department of Pathology, Istanbul University, Istanbul Faculty of Medicine, Istanbul (34390), Turkey

## Abstract

Mucosa associated lymphoid tissue MALT lymphoma is a low grade malignancy that arises most commonly from the gastric mucosa. Small intestinal involvement is very rare. The causative relationship between *Helicobacter pylori and *the gastric MALT lymphoma is a well known issue, but recently there are several data suggesting the role of hepatitis C virus (HCV) infection in the pathogenesis of lymphoma including MALT lymphoma. Herein we present a rare case of duodenal MALT lymphoma with multiple intra-abdominal thromboses together with HCV infection that was confirmed by real-time polymerase chain reaction detecting HCV-RNA within the peripheral blood mononuclear cells.

## Background

Mucosa associated lymphoid tissue (MALT) lymphoma is a neoplasm with low grade malignancy, arising from MALT of various organs including gastrointestinal system, salivary glands, lungs, thyroid glands, thymus, mammary glands and lacrimal glands [1]. Among these sites, MALT lymphoma arising from the gastric mucosa is most frequently reported. Intestinal MALT lymphoma is a very rare neoplasm and there is limited data about clinical and pathological characteristics. The role of *Helicobacter pylori *in the pathogenesis of gastric MALT lymphoma is a well known issue. On the other hand, recently there are some clues that hepatitis C virus (HCV) infection may be a possible contributing infectious factor in the pathogenesis of MALT lymphoma [2,3].

Simultaneous portal, splenic and mesenteric vein thrombosis is a rare condition and may be associated with malignancy, surgery, portal hypertension, hyperhomocysteinemia, Behçet's disease, oral contraceptive use, factor V Leiden mutation, local inflammatory conditions and hematological hypercoagulable states such as paroxysmal nocturnal hemoglobinuria and myeloproliferative disorders [4-6].

Herein we present a case with a history of periodic abdominal pain bouts who admitted with new onset of severe abdominal pain, ascites, signs of malabsorption, and multiple intraabdominal venous thromboses.

## Case Presentation

A 32 year old male presented with severe abdominal pain, fatigue and nausea. He had a seven years history of periodic abdominal pain attacks unrelated to meals and lasting for several days. He had once appendectomy and once laparotomy for acute abdominal findings. Because he had recurrent bouts of abdominal pain, colchicine treatment was started considering the diagnosis of familial Mediterranean fever (FMF). No objective improvement was achieved on this treatment and the patient stopped taking the drug because of the adverse effects such as diarrhea. For the last three weeks, the abdominal pain persisted, became very severe and worsened after meals with nausea and vomiting. In physical examination, the patient was pale and cachectic, abdominal examination revealed guarding and diffuse abdominal tenderness to palpation. There was ascites at level of umbilicus. The patient's past history and family history were noncontributory. In the initial radiological and biochemical assessment, there were no sign of acute abdomen and thus laparotomy was not indicated.

Results of initial laboratory analyses are shown in table 1. Diagnostic paracentesis revealed ascites of portal type (serum-ascites albumin gradient was 2.3 g/dL). The cachexia and decreased levels of transferrin saturation, cholesterol, triglyceride and albumin were assessed as a sign of malabsorption and with these findings we considered a small intestinal disease.

**Table 1:** Results of initial laboratory tests

Complete blood count	Values	References
WBC	7300/uL	(4000-11,000)
Hb	7.4 g/dL	(12-18)
Htc	23.5%	(37-50)
MCV	64.0 fL	(80-100)
Platelet	286000/uL	(150,000-400,000)
**Biochemistry**		
Creatinine	0.8 mg/dL	(0.7-1.4)
Calcium	7.6 mg/dL	(8.5-10.5)
Phosphrus	3.5 mg/dL	(2.7-4.5)
ALP	398 U/L	(30-135)
AST	24 U/L	(5-42)
ALT	20 U/L	(5-45)
LDH	474 U/L	(240-480)
GGT	68 U/L	(5-85)
Total bilirubin	0.60 mg/dL	(0.2-1.0)
Indirect bilirubin	0.31 mg/dL	(0.10-0.50)
Total cholesterol	88 mg/dL	(130-200)
HDL	16 mg/dL	(>40)
LDL	41 mg/dL	(100-130)
Triglyceride	94 mg/dL	(<30)
Total protein	4.6 g/dL	(6.0-8.0)
Albumin	1.99 g/dL	(3.2-5.5)
ESR	42 mm/h	(0-20)
Hs-CRP	143.3 mg/L	(0.0-5.0)

(White blood cells: WBC, Hemoglobin: Hb, Hematocrit:Htc, Mean corpuscular volume:MCV, Alkaline phosphatase:ALP, Aspartat aminotransferase:AST, Alanine aminotransferase:ALT, Lactate dehydrogenase:LDH, Gamma glutamyl transferase:GGT, High density lipoprotein: HDL, Low density lipoprotein:LDL, Erythrocyte sedimentation rate:ESR, high selective C reactive protein: hs-CRP)

**Figure 1:**
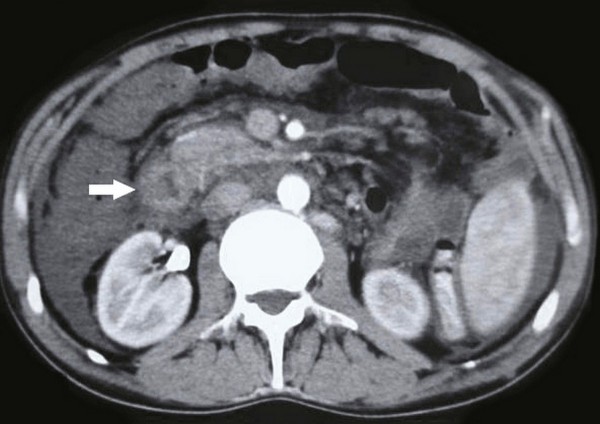
**Late phase contrast enhanced CT without oral contrast demonstrates diffuse wall thickening and narrowing in second part of the duodenum (arrow)**.

**Figure 2:**
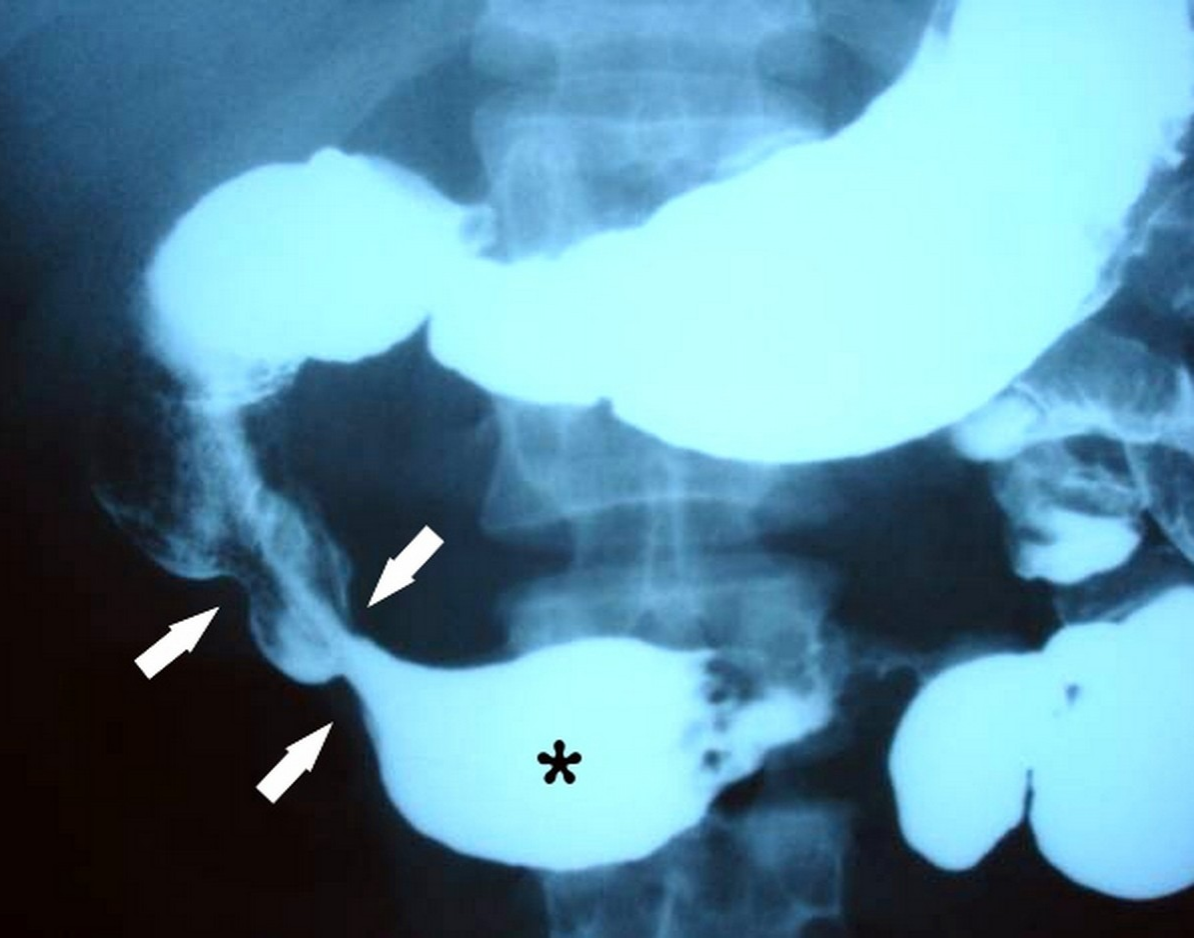
**Upper gastrointestinal series shows multiple narrowing in second part of the duodenum (arrows) and post-stenotic dilatation in third part of the duodenum (asterix)**.

In abdominal ultrasonography (USG), ascites and portal and splenic vein thromboses were observed. Abdominal computed tomography (CT) and CT angiography showed increased wall thickness of intestinal loops, splenic, portal and superior mesenteric vein thrombosis. Arterial pathology was not detected (Figure 1). In small intestinal passage radiograph, 10 cm of descending part of the duodenum, 7 cm of horizontal part of the duodenum and 4 cm of proximal part of the jejunum showed luminal narrowing (Figure 2).

To determine the cause of multiple venous thrombosis in the abdomen, thrombophilic parameters were investigated: Anti-cardiolipin immunoglobulin (IG) G: 4.6 GPLU/mL; anti-cardiolipin IG M: 36.3 MPLU/mL; homocystein (fasting level): 25.1 μmol/L (normal <12 μmol/L); protein C: 94%; protein S: 83%; lupus anticoagulant was negative; factor V Leiden and prothrombin gene mutations were not detected. Immunophenotyping in the peripheral blood revealed normal CD55 and CD59 expression and paroxysmal nocturnal hemoglobinuria (PNH) was not considered. Vasculitis might be responsible for the thrombosis, so rheumatologic diseases were investigated. For Behçet's disease, pathergy test, HLA-B51, HLA-B52 were all negative. Anti-DNA, anti nuclear antibody, anti neutrophil cytoplasmic antibody, anti- Jo-1, anti-RNP, anti-Scl-70, anti-Sm, anti SS-A, anti- SS-B and rheumatoid factor were negative. FMF was considered because of the recurrent bouts of abdominal pain, but available FMF mutation assays were negative. Viral serology was negative for hepatitis B virus (HBV) surface antigen, anti HBV surface antibody and anti-HIV. Anti-HCV (ELISA) was positive but HCV-RNA was negative (RT-PCR method, Roche, COBAS Amplicor HCV monitor kit; linear measurement range: 600-700,000 UI/mL). To investigate possible cryoglobulinemia associated with hepatitis C infection; cryoglobulin and cryofibrinogen tests were performed and found negative.

Because the patient had anti-HCV positivity but HCV-RNA negativity with normal liver transaminases, to confirm the hepatitis C virus infection, HCV-RNA in the plasma and peripheral blood mononuclear cells (PBMC) was tested by RT-PCR method (analytic sensitivity for serum is 20 IU/mL). HCV-RNA was detected in peripheral blood mononuclear cells, so the patient was considered to have hepatitis C virus infection. Liver biopsy revealed non-specific reactive hepatitis and no sign of chronic viral hepatitis, so antiviral treatment was not started.

Malabsorption associated with abdominal pain raised the suspicion of intestinal T cell lymphoma following celiac disease. Anti-gliadin immunoglobulin (Ig) A was 1.2 U/mL and anti-gliadin Ig G was 20.4 U/mL. Anti-endomysium Ig A was negative and serum Ig A level was 163 mg/dL (normal: 68-425 mg/dL), so diagnosis of Celiac disease was not considered.

**Figure 3:**
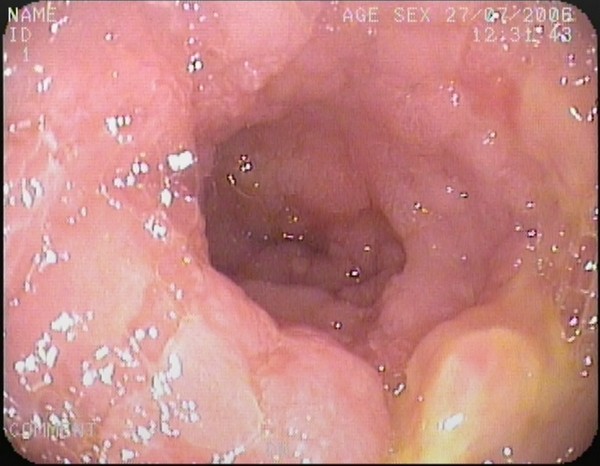
**Endoscopic findings of the descending part of the duodenum**. Mucosa is observed edematous, fragile and granular; multiple small ulcerated lesions are also seen.

**Figure 4:**
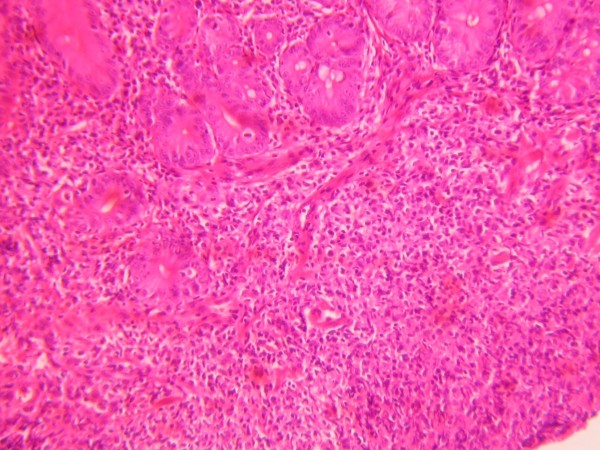
**Endoscopic duodenal biopsy consistent with MALT lymphoma**. Neoplastic infiltration with centrocyte-like morphology that has partially effaced glandular structures in mucosa (HE, ×200)

**Figure 5:**
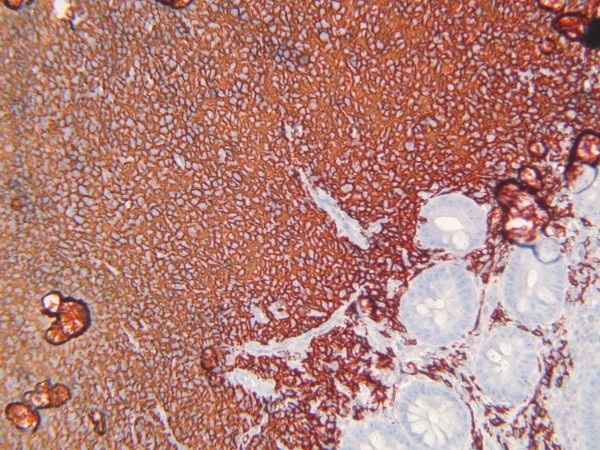
**Endoscopic duodenal biopsy, CD20 immunreactivity in infiltrative population (anti-CD20 antibody clone L-26, AEC chromogene, ×100)**.

In gastroduodenoscopy, duodenum was observed edematous, fragile and granular; multiple small ulcerated lesions were seen (Figure 3). Second part of the duodenum was so narrowed that couldn't be passed through with the endoscope. Multiple duodenal and gastric biopsies were taken during endoscopy. Pathological examination of the duodenal biopsy revealed atypical lymphoid infiltration with centrocyte-like morphology; immunophenotypic investigation was positive for CD 20 (+) and negative for CD 3 and CD 10 which were consistent with the diagnosis of low grade MALT lymphoma of the duodenum (Figure 4, 5). Pathology of gastric biopsies was defined as non-active, non-atrophic chronic gastritis. *Helicobacter pylori *was negative in biopsy specimens. Colonoscopy was also performed and was normal. In bone marrow biopsy, mild hypercellularity was seen and infiltration was not detected.

Diagnosis of MALT lymphoma of the small intestine was considered and COP treatment protocol (cyclophosphamide 750 mg/m^2 ^and 2 mg vincristine for once and 100 mg/day prednisolone for 5 days) was started. Because *Helicobacter pylori *was negative in pathology specimens, eradication therapy was not administrated. At follow up, HCV RNA was still negative and liver function tests remained stable. Because the patient had multiple venous thromboses in abdomen, enoxaparine in treatment dose followed by warfarin was started. After these treatments, patient's complaints and findings of malabsorption were dramatically improved. Control gastroduodenoscopy 4 months after treatment revealed regression of the lesions; endoscopic biopsies were repeated in which no infiltration was detected. Follow up small intestinal passage radiograph was normal.

## Discussion

Primary gastrointestinal (GI) lymphoma accounts for approximately 30% of all extranodal lymphomas [1,7]. However, primary GI lymphomas represent only 1-4% of malignant tumors of the GI tract [8,9]. Sites of involvement include the stomach, small intestine, ileo-cecal area and large intestine. Lymphoma limited to the duodenum is very rare, only a few cases are present in the literature.

The presenting symptoms of duodenal lymphoma are insidious and non-specific and depend on the location of the tumor, the degree of obstruction, and the rapidity of growth. Our case presented with abdominal pain, nausea and vomiting especially just after the meals probably related to intestinal obstruction. He also had signs of malabsorption due to infiltration of the duodenum and jejunum. After successful treatment of lymphoma and nutritional replacement therapy, all these findings subsided.

Predisposing factors for lymphoma of the small intestine include prior malabsorption syndromes (e.g. Celiac sprue and dermatitis herpetiformis); inflammatory bowel disease (e.g. Crohn's disease), acquired immunodeficiency states, *Helicobacter pylori *infection and possibly HCV infection. Infectious agents like *Helicobacter pylori*, *Campylobacter jejuni, Borrelia burgdorferi, Chlamydia psittaci *and HCV indirectly increases the probability of lymphoid transformation by chronically stimulating the immune system to maintain a protracted proliferative state [10].

HCV is both hepatotropic and lymphotropic. Association between HCV infection and mixed cryoglobulinemia, a benign lymphoproliferative disorder is well known but the role of HCV in the pathogenesis of frank lymphoma is not as certain, and is under ongoing discussion, but some authors suggest that HCV may be considered, in addition to *Helicobacter pylori*, as another potential infectious co-factor in the multiple-stage pathogenesis of low-grade lymphomas of MALT type [2].

Isolated anti-HCV positivity in individuals with negative serum HCV-RNA may be related to past infection or false positivity, but this has not yet been completely understood. In our case, anti-HCV was positive but HCV-RNA was negative and liver enzymes were normal. To confirm the diagnosis of hepatitis C virus infection, HCV-RNA was detected by RT-PCR within the PBMCs. Presence of HCV within the lymphoid cells may have contributed to development of intestinal MALT lymphoma, but to our knowledge there is no published report investigating the association of isolated anti-HCV positive hepatitis C virus infection and lymphoma.

After the chemotherapy including corticosteroids, our patient was followed by serial liver enzymes and HCV-RNA tests, but signs of activation of HCV infection did not occur; liver enzymes were always in normal range and HCV-RNA was persistently negative.

One of the unique features of this case is the associated multiple intraabdominal venous thrombosis, including portal vein, splenic vein and superior mesenteric vein. Patients with combination of these thromboses may present with abdominal pain or findings of chronic portal hypertension [5,6]. Our patient had a new onset abdominal pain different in character from the previous attacks which may have been due to intestinal obstruction as well as the thromboses. Known thrombophilic diseases were investigated but no risk factors apart from mild hyperhomocysteinemia could be detected. Such combined thromboses are reported in patients with mild as well as severe hyperhomocysteinemia but generally with other thrombophilic risk factors like essential thrombophilia and factor V Leiden mutation [4].

One of the most important mechanisms of hypercoagulability in malignant diseases is the action of tissue factor, which may be tumor cell-derived [11] or may originate from the tumor-associated environment [12]. In our case the venous thrombosis occurred in the proximity of the tumor and this may be explained by the higher density of the tumor cell-derived pro-thrombotic factors in the neighboring great veins draining the malignant environment. To our knowledge there is no previous report of portal, splenic or superior mesenteric vein thrombosis associated with intestinal MALT lymphoma.

## Conclusion

We have reported a rare case of MALT lymphoma of the duodenum and jejunum together with multiple intraabdominal thromboses and HCV infection proven by detection of HCV-RNA in PBMCs. All patients with MALT lymphoma should be screened for HCV infection. Isolated anti-HCV positivity should be confirmed by detection of HCV-RNA in PBMCs to exclude false positivity, because diagnosis of HCV infection is important in follow up of patients who are given immunosuppresive chemotherapeutics for lymphoma. HCV infection may play a role in the pathogenesis of MALT lymphoma beside *Helicobacter pylori *and antiviral treatment for HCV may cause regression of the MALT lymphoma and may be used for its treatment similar to *Helicobacter pylori *eradication therapy. Pathophysiological link between HCV infection and MALT lymphoma and its impact on the treatment should be further investigated.

## Consent

Written informed consent was obtained from the patient for publication of this case report and accompanying images. A copy of the written consent is available for review by the journal's Editor-in-Chief.

## Competing interests

The authors declare that they have no competing interests.

## Authors' contributions

AO: Follow-up of the patient and writing the manuscript.

FT: Follow-up of the patient and writing the manuscript.

SN: Follow-up of the patient and writing the manuscript.

MB: Forming and explanation of the radiological figures.

BP: Providing the explanation of the endoscopic findings.

OD: Forming and explanation of the pathology figures.

MAK: Follow-up of the patient and writing the manuscript.

CT: Follow-up of the patient and writing the manuscript.

All authors read and approved the final manuscript.
